# Detection of *Mycobacterium tuberculosis* in pediatric stool samples using TruTip technology

**DOI:** 10.1186/s12879-019-4188-8

**Published:** 2019-06-27

**Authors:** Annelies W. Mesman, Martin Soto, Julia Coit, Roger Calderon, Juan Aliaga, Nira R. Pollock, Milagros Mendoza, Francisco M. Mestanza, Carlos J. Mendoza, Megan B. Murray, Leonid Lecca, Rebecca Holmberg, Molly F. Franke

**Affiliations:** 1 Department of Global Health and Social Medicine, Harvard Medical School, Boston, USA; 2 Socios En Salud Sucursal (Partners In Health), Lima, Peru; 3 Department of Laboratory Medicine, Boston Children’s Hospital, Boston, USA; 4 Ministerio de Salud del Peru, Jesús María, Peru; 5 Akonni Biosystems Inc, Frederick, USA

**Keywords:** Tuberculosis, Children, Molecular diagnostics, TruTip

## Abstract

**Background:**

Rapid and accurate diagnosis of childhood tuberculosis (TB) is challenging because children are often unable to produce the sputum sample required for conventional tests. Stool is an alternative sample type that is easy to collect from children, and studies investigating the use of stool for molecular detection of *Mycobacterium tuberculosis (Mtb)* have led to promising results. Our objective was to evaluate stool as an alternative specimen to sputum for *Mtb* detection in children. We did so using the TruTip workstation (Akonni Biosystems), a novel automated lysis and extraction platform.

**Methods:**

We tested stool samples from 259 children aged 0–14 years old, in Lima, Peru who presented with TB symptoms. Following extraction with TruTip, we detected the presence of *Mtb* DNA by IS6110 real-time PCR. We calculated assay sensitivity in two groups: (1) children with culture confirmed TB (*N* = 22); and (2) children with clinically-diagnosed unconfirmed TB (*N* = 84). We calculated specificity among children in whom TB was ruled out (*N* = 153). Among children who were diagnosed with TB, we examined factors associated with a positive stool test.

**Results:**

Assay sensitivity was 59% (95% confidence interval [CI]: 39–80%) and 1.2% (95% CI: 0.0–6.5%) in children with culture-confirmed and clinically-diagnosed unconfirmed TB, respectively, and specificity was 97% (95% CI: 93–99%). The assay detected *Mtb* in stool of 7/7 children with smear-positive TB (100% sensitivity; 95% CI: 59–100%), and in 6/15 of children with smear-negative, culture-confirmed TB (40% sensitivity; 95% CI: 16–68%). Older age, smear positivity, culture positivity, ability to produce sputum and cavitary disease were associated with a positive stool result.

**Conclusion:**

Testing of stool samples with the TruTip workstation and IS6110 amplification yielded sensitivity and specificity estimates comparable to other tests such as Xpert. Future work should include detection of resistance using the TruTip closed amplification system and assay optimization to improve sensitivity in children with low bacillary loads.

## Background

The World Health Organization estimates that one million new pediatric tuberculosis (TB) cases and 194,000 childhood TB deaths occurred in 2017 [1]. Rapid case detection and treatment initiation is critical to minimizing TB morbidity and mortality in children but is hampered by the absence of a rapid, accurate diagnostic tool for this group. Bacteriologic confirmation of *Mycobacterium tuberculosis (Mtb*) in children is often difficult to achieve because they are frequently unable to expectorate sputum for bacteriologic testing and often have paucibacillary disease that cannot be detected using sputum smear microscopy, culture, and/or molecular testing [e.g. Xpert (Cepheid, Sunnyvale CA, USA)]. Sputum induction and gastric aspiration can be used to obtain respiratory specimens from children unable to expectorate sputum; however, gastric aspiration is invasive and neither procedure is widely implemented in resource-constrained settings. Due to these diagnostic challenges, bacteriologic confirmation of TB is obtained in only a small minority of children diagnosed with TB [2–4].

Stool can be easily obtained from most children and *Mtb* can be detected in stool using Xpert [5–10] or other laboratory-developed PCR assays [11–13]. In the case of Xpert, the process is automated, but detection of drug resistance is currently limited to rifampin-associated resistance mutations in rpoB. Non-integrated methods for DNA isolation and amplification using extraction kits and in-house tests are often laborious, multistep procedures. An ideal test would be an automated point-of-care workstation with integrated capacity for both *Mtb* and expanded drug resistance testing-criteria listed in the target product profile for novel TB diagnostics in low resource settings [14]. The TruTip workstation is an automated platform including lysis and homogenization with TruTip nucleic acid extraction and purification (Akonni Biosystems, Frederick, MD, USA) [15–17]. TruTip has been used for nucleic acid isolation from a variety of pathogens and sample types and has demonstrated efficient *Mtb* DNA recovery from raw sputum [15, 18]. The platform can be connected to a closed amplicon system for amplification and microarray-based detection of *Mtb* as well as a number of drug resistance-associated mutations [17, 19–21]. The aim of the present study was to estimate sensitivity and specificity of *Mtb* detection in stool from children with symptoms compatible with intrathoracic TB in Lima, Peru, using this novel technology with IS6110 real-time PCR.

## Methods

### Ethics

Study participants’ guardians provided written informed consent to participate, and children eight years of age and older provided written assent. Consent for publication was not applicable. All study procedures were approved by the Ethics Committee of Peru’s National Institute of Health and the Office of Human Research Administration at Harvard Medical School.

### Study population

Between May 2015 and February 2018, we recruited children to participate in a pediatric TB diagnostics study. Eligible children were less than 15 years of age, had a history of contact with an adult with TB within the previous two years, and presented to a participating public sector health center in Lima, Peru with symptoms compatible with TB (i.e., persistent cough for more than two weeks; unexplained weight loss; unexplained fever for more than one week; and/or unexplained fatigue or lethargy) [22]. For this analysis, we included the subset of children with culture-confirmed TB or clinically-diagnosed unconfirmed TB who had at least one stool sample available. For each case, we selected up to two children in whom TB had been ruled out (i.e, controls), matching on age and sample collection date when possible.

### Study procedures and sample collection

Children were evaluated for TB per Peruvian National Tuberculosis Strategy guidelines [23]. In brief, children provided up to two gastric aspirate (GA) and/or sputum samples (expectorated or induced) for smear and culture, and Ministry of Health pediatric pulmonologists considered these results as well as medical history, physical examination, chest X-ray findings and tuberculin skin testing (TST) results to diagnose or rule out TB. GA samples were neutralized to a pH of 6.8–7.2 upon collection. We requested two stool samples from all children for research purposes. From children who were diagnosed with TB, we aimed to collect these samples prior to TB treatment initiation. Stool collection took place at home or the health center. For children in diapers, plastic wrap was inserted on the inside of the diaper and a urine collection bag was adhered to the child. The latter served to collect a urine sample as well as to prevent mixing of urine and stool. Following collection, all samples were transported by cold-chain to the laboratory, aliquoted and stored at − 80 °C until testing. Because testing of stool did not occur in real time and because the assay was experimental, stool testing results were not shared with families or healthcare providers. HIV testing was not performed because the HIV prevalence among children in the study setting is low (< 0.1%) [24].

### Laboratory procedures

Sample processing and testing was performed in the BSL-3 and BSL-2 facilities at the Socios En Salud Sucursal Laboratory in Lima. We centrifuged and decontaminated sputum and GA samples in 2% NaOH/0.25% n-acetyl-L-cysteine (NALC). GA and sputum samples were analyzed by Ziehl-Neelsen staining/smear microscopy and liquid culture (BACTEC MGIT 960, BD Franklin Lakes, USA).

For lysis and DNA extraction procedures using the TruTip workstation we followed methods similar to those previously described for sputum samples [18]. This TruTip protocol performed best in a head-to-head comparison to five other technologies for bacterial nucleic acid extraction from stool [25]. In brief, 500 mg of thawed stool was homogenized in TE buffer in a magnetically-induced vortexing (MagVor) element, and after a 20 min heating step at 56 °C, the samples were transferred to the TruTip workstation for DNA extraction [16]. *M*t*b* DNA was amplified in duplicate by real-time PCR using a Roche 480 Lightcycler (Basel, Switzerland) with primers targeting the insertion element IS6110 [26]. We considered a sample positive for *Mtb* DNA if, for both PCR replicates, the cycle threshold (Ct) value was < 37 and the change in fluorescence was > six units. We repeated the PCR step in duplicate for discordant replicates and considered the sample to be indeterminate for *Mtb* DNA if the two replicates remained discrepant after a second PCR run. Laboratory staff were blinded to participants’ clinical status.

### Data analysis

Data were analyzed using SAS (Version 9.4. SAS Institute Inc., Cary, NC, USA). A child was classified as having confirmed TB if s/he had a positive culture result from sputum or GA and having clinically-diagnosed unconfirmed TB if a physician made a TB diagnosis in the absence of a positive culture result. We calculated sensitivity and specificity and 95% confidence intervals (CI) both at the child-level (including all samples from each child) and at the sample-level. Sensitivity of *Mtb* detection from stool was calculated separately for children with confirmed TB and clinically-diagnosed unconfirmed TB. Specificity was estimated among those in whom TB had been ruled out. In per-sample analyses we adjusted confidence intervals for clustering from multiple samples per child. Among all children with a TB diagnosis, we used chi-square tests to examine the associations between a positive stool test and the following variables: age, sex, smear and culture results, ability to spontaneously expectorate sputum, and cavitation on chest x-ray. Numerical precision for all percentages, *p*-values and 95% CIs are presented based on the evidence-based recommendations proposed by Cole [27].

## Results

### Study population

**Fig. 1:**
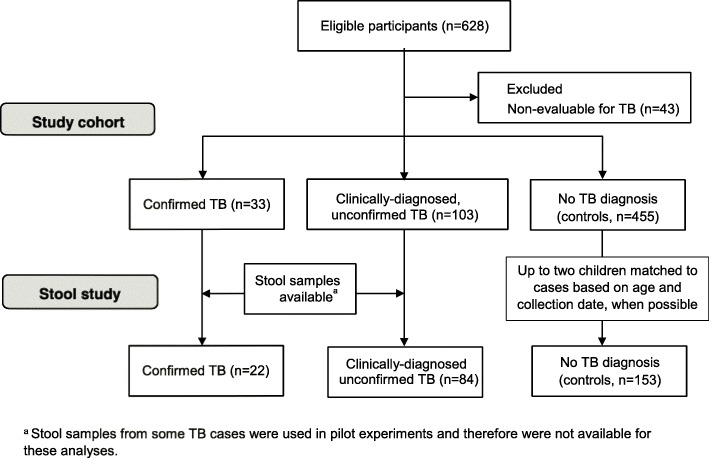
Flowchart of cohort enrollment and stool study inclusion

From 628 children enrolled in the study, we selected 259 children based on stool sample availability (22 children with culture confirmed TB, 84 children with clinically-diagnosed unconfirmed TB, and 153 children in whom TB had been ruled out (Fig. 1)). These children had a median age of 5.1 years and 48% were female. Nearly all children (255/259; 98%) had at least one respiratory sample collected. Of the four with no respiratory sample, three were diagnosed with TB based on clinical criteria and TB was ruled out in one. Children between the ages of 11 and 14 years old were more likely than younger children to have a culture-confirmed diagnosis, and seven children had a smear-positive sputum sample, with AFB results of ‘+’ (one child) ‘++’ (two children), or ‘+++’ (four children). All seven children with smear-positive sputum belonged to the older age group (Table 1). A minority of children (41%) were able to spontaneously expectorate a sputum sample for culture. In contrast, the majority (59%, 13/22) of children with confirmed TB, and all children with smear-positive disease, were diagnosed based on analyses of expectorated sputum (Table 1).

**Table 1:** Demographic and clinical characteristics of children, stratified by TB status

Variable		All children (*N* = 259) *n* (%)	SM+, CU+ TB^a^ (*N* = 7) *n* (%)	SM-, CU+ TB^a^ (*N* = 15) *n* (%)	All CU+ TB (*N* = 22) *n* (%)	Clinically-diagnosed unconfirmed TB (*N* = 84) *n* (%)	No TB diagnosis (*N* = 153) *n* (%)
Age group (years)	0–4	125 (48)	0 (0)	8 (53)	8 (36)	42 (50)	75 (49)
	5–10	37 (14)	0 (0)	5 (33)	5 (23)	34 (40)	58 (38)
	11–14	97 (37)	7 (100)	2 (13)	9 (41)	8 (10)	20 (13)
Female sex		124 (48)	5 (71)	6 (40)	11 (50)	47 (56)	66 (43)
Ability to spontaneously produce a sputum sample		106 (41)	7 (100)	6 (40)	13 (59)	32 (38)	61 (40
Cavitary disease on chest x-ray		5 (1.9)	5 (71)	0 (0)	5 (23)	0 (0)	0 (0)

^a^*CU* culture, *SM* smear

From the 259 children, we collected a total of 484 stool samples. Most children had two stool samples; however 34 children (13%) had only one sample available. Seventeen samples from children diagnosed with TB were collected after TB treatment initiation (median time from treatment initiation to collection: three days; range [1–11 days]). Eight samples were collected 72 h or more after treatment start. Four of these eight samples were from children with smear-positive sputum. This occurred because children with a positive sputum smear result were eligible to initiate TB treatment immediately, and did not need to await culture results. This resulted in a narrow window between enrollment and treatment initiation during which pre-treatment samples could be collected.

### Per sample analyses

The assay detected *Mtb* in 25/484 samples. Only one sample, from a child in the control group, had an indeterminate result, which we excluded from further analyses. Among 13 stool samples from seven children with smear positive sputum, 100% (95% confidence interval [CI]: 59–100%) were positive by the stool assay (Table 2). Sensitivity was lower in the smear-negative culture-confirmed group (23%; 95% CI: 5.6–41%, Table 2). We detected *Mtb* in 4/282 (1.4%) samples from control children, resulting in a specificity of 99% (95% CI: 97–99.8%).

**Table 2:** Per sample analyses of *Mtb* stool assay in 259 children

	Positive stool result (n/N)	Indeterminate stool result (n/N)	Sensitivity (95% CI^a^)	Specificity (95% CI^a^)
All samples	25/484	1/484		
Confirmed TB (SM+)^b^	13/13	0/13	100% (59–100%)	
Confirmed TB (SM-)	7/30	0/30	23.3% (5.6–41%)	
Confirmed TB (all smear grades)	20/43	0/43	47% (27–66%)	
Clinically-diagnosed, unconfirmed TB	1/159	0/159	0.6% (0.00–1.9%)	
No TB diagnosis	4/282	1/282		99% (97–100%)

^a^*CI* confidence interval; 95% CI are adjusted for multiple samples per patient. ^b^*SM* smear

### Per child analyses

In child-level analyses, sensitivity of the stool assay conducted in each child’s first sample was 55% (95% CI: 33–75%). When we included the results of both samples, we identified one additional confirmed TB case, which increased sensitivity to 59% (95% CI: 39–80%) (Table 3). While the majority of children with a positive stool sample also had a spontaneous sputum sample that was culture positive (11/14, 79%), we detected *Mtb* in the stool of two children in whom TB was confirmed based on gastric aspirate and one child with clinically-diagnosed unconfirmed TB (this child had one expectorated and one induced sputum sample, both of which tested negative for *Mtb*). All three children were under five years of age.

**Table 3:** Child-level analyses of *Mtb* stool assay in 259 children

		Positive stool result (n/N)	Sensitivity (95% CI^a^)	Specificity (95% CI)
First sample	All children	16/259		
	Confirmed TB (SM+)^b^	7/7	100% (59–100%)	
	Confirmed TB (SM-)	5/15	33% (13–61%)	
	Confirmed TB (all smear grades)	12/22	55% (33–75%)	
	Clinically-diagnosed, unconfirmed TB	1/84	1.2% (0.0–6.5%)	
	No TB diagnosis	3/153		98% (94–99.5%)
Both samples	All children	18/259		
	Confirmed TB (SM+)	7/7	100% (59–100%)	
	Confirmed TB (SM-)	6/15	40% (16–68%)	
	Confirmed TB (all smear grades)	13/22	59% (36–79%)	
	Clinically-diagnosed, unconfirmed TB	1/84	1.2% (0.0–6.5%)	
	No TB diagnosis	4/153		97% (93–99.3%)

^a^*CI* confidence interval, ^b^*SM* smear

### Factors associated with stool test positivity among TB cases

Features of adult-like TB (i.e., a positive smear result, a positive culture result, the ability to spontaneously expectorate sputum and cavitary TB), were associated with detection in stool and more common in the 11–14 year age group (Table 4). There was no association between stool positivity and sex.

**Table 4:** Factors associated with *Mtb* stool assay positivity among children with culture-confirmed or clinically-diagnosed, unconfirmed TB (*N* = 106)

Variable		Positive stool result (*N* = 14) *n* (%)	Negative stool result (*N* = 92) *n* (%)	P-value
Age group (years)	0–4	3 (21)	47 (51)	0.0008^a^
	5–10	4 (29)	35 (38)	
	11–14	7 (50)	10 (11)	
Female sex		7 (50)	51 (55)	0.8^b^
Positive smear result		7 (50)	0 (0.0)	< 0.0001^b^
Positive culture result		13 (93)	9 (9.8)	< 0.0001^b^
Ability to spontaneously produce a sputum sample		12 (86)	33 (36)	0.0006^a^
Cavitary disease on chest x-ray		5 (36)	0 (0.0)	< 0.0001^b^

^a^Chi-square test ^b^Fisher’s Exact test

## Discussion

We found that our test was 100% sensitive for *Mtb* detection in stool among children with smear-positive confirmed TB and 40% sensitive in children with smear-negative confirmed TB. Importantly, we detected *Mtb* in stool of three children below the age of five who were not able to expectorate sputum (two of whom were diagnosed based on GA samples and one who was culture negative by sputum, but clinically-diagnosed).

Our study of 259 children included 106 (40%) TB cases, of whom 22 (21%) had culture-confirmed TB, reflecting the poor sensitivity of culture for pediatric TB. These numbers are similar to the number of culture-confirmed children in other studies [6, 10, 28–33]. The sensitivity (59%) and specificity (97%) of this stool assay relative to liquid culture of respiratory samples was comparable to previous studies using Xpert for stool testing in children [7]. Sensitivity of Xpert stool testing among children was estimated 67% (95% CI: 52–79) in a recent systematic review. The authors highlighted the heterogeneity in design between studies as contributor to the wide variability in sensitivity, which ranged from 32 to 85%, but did not stratify for smear results [34].

Unfortunately, we detected TB in only 1.2% of children with culture negative, clinically-diagnosed TB. Other studies have reported similarly low sensitivities in children with clinically-diagnosed unconfirmed TB, ranging from 0 to 3% [6, 28, 29, 35]. Considerations for improving sensitivity include incorporating an initial homogenization step prior to aliquotting, testing of multiple sample aliquots, incorporating inhibitor-removal compounds, and altering the sample volume. Our stool test protocol required a relatively small input volume (500 mg), which may minimize the impact of PCR inhibitors in stool that can impede the detection of *Mtb*. In fact, several studies show reduced sensitivity with larger sample volumes. For example, Banada et al. reported higher sensitivity and fewer invalid test results when stool input volume was decreased from 1200 to 600 mg [5]. Recently, DiNardo et al. obtained 67% sensitivity with an in-house developed method using only 50 mg, demonstrating that even smaller volumes can be sufficient to reach high sensitivity. Our assay had a limit of detection of at least 80 CFU/ml for sputum [18]; future work will include optimization steps described above and determination of the limit of detection for the stool application.

In line with previous reports [8, 36] we found strong associations between a positive stool test and features of more adult-like TB (i.e., cavitary disease; positive sputum culture; positive sputum smear; the ability to produce sputum), which were most common in the oldest age group. This emphasizes the impact of age and smear status on the outcomes of stool testing and has critical implications for the reporting of pediatric TB diagnostic studies because stool assay sensitivities may vary widely based on the percentage of children in the cohort with adult-like TB and/or high bacterial burden. Because children with adult-like TB are often able to produce a sputum sample for testing, a stool assay will only begin to fill the pediatric TB diagnostic gap if it is sensitive among children who cannot already spontaneously expectorate sputum. To facilitate an understanding of stool assay performance and its utility across settings, we recommend stratifying stool sensitivity results by some measure of disease severity, ideally smear status, and reporting the added yield among children unable to spontaneously expectorate sputum.

Akonni TruTip is not yet commercially produced; we expect the per-assay cost in combination with PCR detection to be competitive with existing TB diagnostic assays, such as Xpert. The TruTip workstation had a number of attractive attributes for *Mtb* DNA extraction from stool. Extraction procedures required limited pipetting steps compared to commercially available kits. Additional future advantages include that the extracted DNA eluate can be isolated for other purposes, such as sequencing. Most importantly, the TruTip workstation can be integrated with a microarray-based detection of *Mtb* as well as a number of drug resistance-associated mutations [17, 19, 20], which is a focus of future studies.

A limitation of our study was that we lacked follow-up data on the clinical evolution of children, a key criterion for classifying pediatric cases of clinically-diagnosed, unconfirmed TB. To the extent that unconfirmed clinical TB was overdiagnosed, sensitivity will be underestimated in this group. Similarly, missed TB diagnoses among children in whom TB was ruled out could lead to an underestimate of assay specificity. The influence of these potential biases appears limited given the very low sensitivity among children with clinically-diagnosed unconfirmed clinical TB and a high overall specificity. A second limitation of our study is the absence of children living with HIV. Studies have shown that sensitivity of stool assays may be higher in children living with HIV [6, 7], in particular with severe immunosuppression [30], therefore, our results may not be generalizable to children living with HIV.

## Conclusion

In conclusion, the results of our study show that we can detect *Mtb* in pediatric stool samples. We used the TruTip workstation in combination with realtime IS6110 PCR for detection, and its performance is comparable to other platforms. Future work should include detection of resistance in stool samples using the TruTip closed amplification system and assay optimization to improve sensitivity in children with low bacillary loads.

## Data Availability

Reported data are available on request to the corresponding author.

## Abbreviations

AFB: Acid fast bacilli
Ct: Cycle threshold
DST: Drug sensitivity testing
Mtb: *Mycobacterium tuberculosis*
PCR: Polymerase Chain Reaction
TB: Tuberculosis
TST: Tuberculin skin test
WHO: World Health Organization
