# Correction to: Detection of *Mycobacterium tuberculosis* in pediatric stool samples using TruTip technology

**DOI:** 10.1186/s12879-019-4483-4

**Published:** 2019-10-16

**Authors:** Annelies W. Mesman, Martin Soto, Julia Coit, Roger Calderon, Juan Aliaga, Nira R. Pollock, Milagros Mendoza, Francisco M. Mestanza, Carlos J. Mendoza, Megan B. Murray, Leonid Lecca, Rebecca Holmberg, Molly F. Franke

**Affiliations:** 1 Department of Global Health and Social Medicine, Harvard Medical School, Boston, USA; 2 Socios En Salud Sucursal (Partners In Health), Lima, Peru; 3 Department of Laboratory Medicine, Boston Children’s Hospital, Boston, USA; 4 Ministerio de Salud del Peru, Jesús María, Peru; 5 Akonni Biosystems Inc, Frederick, USA

**Correction to: BMC Infect Dis**

**http://dx.doi.org/10.1186/s12879-019-4188-8**

Following publication of the original article [1]. The authors reported that there is a mistake in Fig. 1: the number of patients in the control group its 449 patients, instead of 455.

**Fig. 1:**
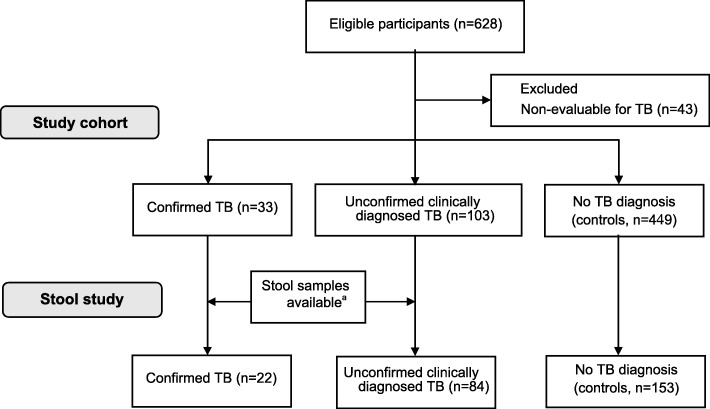
Flowchart of cohort enrollment and stool study inclusion

The correct version of Fig. 1 can be found below.
